# Recurrent superior orbital fissure syndrome associated with VEXAS syndrome

**DOI:** 10.1186/s12348-023-00362-1

**Published:** 2023-09-07

**Authors:** Katie Myint, Namritha Patrao, Oana Vonica, Kaveh Vahdani

**Affiliations:** 1 Adnexal Service, Moorfields Eye Hospital NHS Foundation Trust, London, UK; 2 Royal Eye Unit, Kingston Hospital NHS Foundation Trust, Surrey, UK

## Abstract

**Purpose:**

To describe a case of recurrent orbital inflammation and superior orbital fissure syndrome associated with VEXAS (vacuoles, E1 enzyme, X-linked, auto-inflammatory, somatic) syndrome.

**Case presentation:**

VEXAS syndrome is a recently identified multi-system inflammatory disease of late adult onset. The authors describe the case of a 76-year-old man who presented with recurrent episodes of orbital inflammation, with superior orbital fissure syndrome, dacryoadenitis and orbital myositis. He had a constellation of systemic disorders including recurrent chest infections, congestive cardiac failure, pulmonary emboli and skin rashes. The underlying diagnosis of VEXAS syndrome was confirmed by genetic testing, which revealed the UBA1 mutation.

**Conclusion:**

VEXAS syndrome should be considered in the differential diagnosis of orbital inflammatory disease associated with multi-system inflammatory disorders.

## Introduction

First described by Beck et al. in 2020, VEXAS (vacuoles, E1 enzyme, X-linked, auto-inflammatory, somatic) syndrome is a rare and potentially lethal disease characterized by multi-system inflammatory and or hematological disorders caused by somatic mutations in ubiquitin activating enzyme 1 (UBA1) in hematopoietic progenitor cells [1]. UBA1 is an X-linked gene that encodes E1 activating enzyme, which is essential for ubiquitylation-dependent intracellular protein degradation and cell homeostasis. In VEXAS syndrome, a somatic mutation (acquired later in life) in UBA1 in hematopoietic progenitor cells leads to reduced cytoplasmic expression of E1 activating enzyme. As a result, there is upregulation of inflammatory cytokines such as IFN-γ, IL-8 and IP-10, causing systemic inflammation with multi-organ involvement. The majority of patients with VEXAS syndrome are older men, with a median age of 74 years at diagnosis [2]. Rarely, VEXAS syndrome has also been reported in women, which is thought to arise through X-inactivation. Genome-driven studies report the prevalence of disease-causing UBA1 variants as 1 in 4269 males older than 50 years, and 1 in 26 238 women older than 50 years [3]. As the condition has only recently been identified, patients may be diagnosed months to years after symptom onset. In one notable case, a patient was diagnosed with VEXAS posthumously, 8 years after his first symptoms [4].

Common clinical features in VEXAS syndrome include arthritis, relapsing polychondritis, vasculitis, cytopenia and macrocytic anemia, in the presence of raised inflammatory markers and constitutional symptoms such as fatigue, weight loss and fevers [2]. Various ophthalmic manifestations have also been documented including ocular, periorbital and orbital inflammatory syndromes. The authors describe a case of recurrent orbital inflammation with superior orbital fissure syndrome, dacryoadenitis, and orbital myositis associated with VEXAS syndrome, and review the literature on VEXAS syndrome ophthalmic manifestations.

## Case presentation

A 76-year-old man was referred with recurrent episodes of right orbital inflammation, each lasting for less than a week before spontaneously resolving. He had no medical history of note 6 months prior to his ophthalmic symptoms, when he developed unexplained intermittent pyrexia, recurrent chest infections, congestive cardiac failure and thromboembolic events (Table 1), requiring multiple hospitalizations including intensive care. Six months later, he presented with complete right blepharoptosis, preceded by 5 days of painless right upper lid swelling and redness, and partial ophthalmoplegia (elevation, adduction, and abduction deficit). Magnetic resonance imaging of the head and orbits was unremarkable, and the eyelid swelling completely resolved within few days without treatment, although there was mild residual blepharoptosis.

**Fig. 1:**
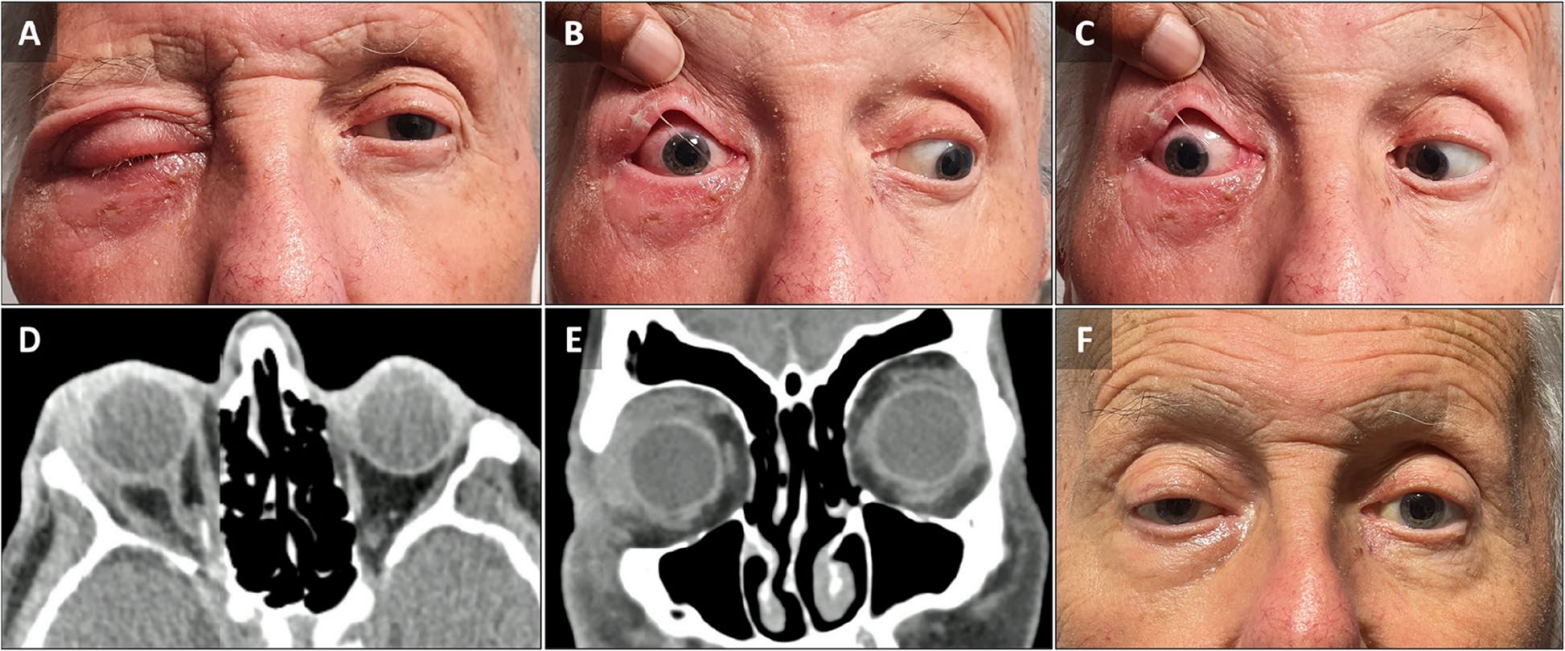
Clinical and radiological findings at the time of symptom recurrence. **A** Right periorbital swelling and ptosis. **B**, **C** Conjunctival injection and ophthalmoplegia. **D** axial CT imaging of the orbits demonstrates relative right proptosis, anterior orbital soft tissue swelling, enlarged lacrimal gland, mild enlargement of the medial and lateral rectus muscles, subtle nerve sheath thickening, thickening of the anterior cavernous sinus (arrow) and soft tissue changes within superior orbital fissure/orbital apex. **E** Coronal CT orbit demonstrating enlarged right lacrimal gland with spill-over inflammation into superior rectus and levator muscle complex. **F** Residual mild right ptosis after resolution of orbital inflammation

Two months later, he presented with severe right ophthalmoplegia, proptosis, periorbital edema and conjunctival chemosis (Fig. 1A-C), with visual acuities of 20/50 in the right eye and 20/32 in the left eye, normal pupillary reactions, and no signs of intraocular inflammation. Computed tomography of the orbits demonstrated soft tissue swelling in the anterior orbit, enlargement of the right lacrimal gland and extraocular muscles (notably the levator palpebrae superioris/superior rectus complex and lateral rectus) (Fig. 1D, E), intraconal fat stranding, subtle thickening of the optic nerve sheath, enlargement of the anterior right cavernous sinus, and soft tissue changes within the superior orbital fissure and orbital apex (Fig. 1D), but no discrete collection or paranasal sinus opacification. The orbital inflammation improved significantly after one week of systemic antibiotics (Fig. 1F), but there was mild residual right relative proptosis (1-2 mm), moderate blepharoptosis, and a slight right abduction and elevation, which remained stable at one-year follow up.

Meanwhile, he was undergoing investigations for a suspected multi-organ inflammatory disorder (summarized in Table 1). DNA analysis from bone marrow revealed a mutation in UBA1, confirming the diagnosis of VEXAS syndrome. He was treated with prednisolone (starting with 20 mg daily, reducing by 2.5 mg each week to zero) and hydroxychloroquine 200 mg daily. At one-year follow-up, his ophthalmic condition remained stable, with no further recurrence.

**Table 1:** Systemic manifestations of VEXAS syndrome in a case of a 76-year-old man

Organ system	Symptoms
Respiratory	Recurrent chest infections Pleural effusions Pulmonary hypertension
Cardiovascular	Heart failure with preserved ejection fraction
Haematological	Bilateral pulmonary emboli Saphenous vein occlusion Iron deficiency anemia
Renal	Hyponatremia Microalbuminuria
Skin	Widespread erythematous plaques Leucocytoclastic vasculitis
Musculoskeletal	Muscle wasting Globally stiff movements around the spine and hips
Other	Fatigue Weight loss

## Discussion

Ophthalmic involvement has been observed in up to 40% of VEXAS syndrome patients, with episcleritis, scleritis, and uveitis being the most common manifestations, and orbital and periorbital inflammation occurring less frequently.

A literature review for English language publications with the keyword “VEXAS” produced 85 results, of which 21 articles described ocular features in VEXAS syndrome (summarized in Table 2**)**. Periorbital edema is the most common ocular adnexal finding, with other manifestations including orbital inflammation or cellulitis, orbital myositis, dacryoadenitis, optic perineuritis, or oculomotor nerve paresis.

**Table 2:** Summary of ocular features reported in VEXAS syndrome in the current literature

Author	Number of cases	Demographics		Ocular features
		Sex	Age (years) (median and range)	
Georgin-Lavialle S et al. [5]	116	111 male (96%)	67	Uveitis = 11 (9.5%) Scleritis = 10 (8.6%) Episcleritis = 14 (12.1%) Orbital mass = 4 (3.4%)
Barba T et al. [6]	1	Female	51	Episcleritis
Staels F. et al. [7]	2	2 male (100%)	69, 75	Episcleritis = 2 (100%)
van der Made CI et al. [8]	12	12 male (100%)	67 (47–79)	Posterior scleritis = 1 Blepharitis = 2 Anterior uveitis = 2 Exophthalmos caused by periorbital and infraorbital panniculitis =1
Ciferska H. et al. [9]	3	3 male (100%)	74 (68–76)	Sclerouveitis = 1 Episcleritis = 1
Khitri MY et al. [10]	55	53 male (96%)	66	Uveitis = 9 (17%) Scleritis = 7 (13%) Episcleritis = 15 (28%) Retinal vasculitis = 2 (4%)
Neupane K et al. [11]	1	Male	Early 60s	Scleritis Choroidal effusion Serous retinal detachment Intraretinal haemorrhage
Topilow JS et al. [12]	1	Male	57	Orbital myositis (medial rectus)
Holmes A et al. [13]	1	Male	70	Uveitis Periorbital oedema
Templé M et al. [14]	2	2 male (100%)	74, 71	Retinal vasculitis = 1 (50%)
Al-Hakim A et al. [15]	4	4 male (100%)	56 (49–64)	Periorbital oedema = 1 (25%)
Ciprian G [16]	1	Male	56	Orbital inflammation Scleritis Orbital myositis (superior rectus)
Lacombe V et al. [17]	6	6 male (100%)	74 (70–78)	Scleritis or episcleritis = 2 (33.3%) Periorbital oedema = 2 (33.3%)
Magnol M et al. [18]	1	Male	57	Anterior uveitis
Kunishita Y et al. [19]	3	3 male (100%)	69 (66–73)	Scleritis = 2 (66.7%)
Lee SMS et al. [20]	1	Male	69	Pseudomembranous conjunctivitis Anterior scleritis
Lötscher F et al. [21]	1	Male	68	Scleritis
Goyal A et al. [22]	1	Male	64	Orbital myositis (medial rectus) Periorbital oedema
Midtvedt Ø et al. [4]	1	Male	Late 60s	Iridocyclitis
Beecher M et al. [23]	1	Male	68	Upper lid swelling and oedema Enlarged lacrimal glands
Martín-Nares E et al. [24]	1	Male	77	Orbital and periorbital inflammation Orbital myositis (medial and inferior recti) Epiphora Eye pain Chemosis

Systemic corticosteroids are the mainstay of management for VEXAS syndrome, [2] including its ophthalmic manifestations, although orbital involvement may be self-limiting, as observed in our case. Various steroid-sparing medications, such as mycophenolate and methotrexate, have been shown to be beneficial in the long-term management of VEXAS syndrome. Tocilizumab (a monoclonal IL-6 receptor inhibitor) and ruxolitinimib (a JAK inhibitor) have also been found to be effective in controlling the disease [25]. Azacitidine (a hypomethylating agent) has been reported to be effective in selected patients with VEXAS and associated myelodysplastic syndrome [26]. Furthermore, there have been promising results following allogeneic hematopoeitic stem cell transplant, with some patients achieving complete disease remission [2].

The current case represents a rare ophthalmic manifestation of VEXAS syndrome, with recurrent orbital and anterior cavernous sinus/superior orbital fissure syndrome, orbital myositis, and dacryoadenitis; the ophthalmoplegia being probably caused by a combination of myositic and paralytic factors. Despite the self-limiting inflammatory syndrome and long-term immunosuppressive therapy, the patient had mild persistent ophthalmoplegia and blepharoptosis.

When orbital inflammatory syndrome secondary to systemic disease is suspected, a detailed history, systemic review, and laboratory evaluations are paramount. This approach includes a full blood count, metabolic panel, thyroid function tests, inflammatory markers, and an autoimmune profile encompassing anti-nuclear antibodies (ANA), antineutrophil cytoplasmic antibodies (ANCA), serum angiotensin-converting enzyme (ACE) and immunoglobulin G4 (IgG4) levels. CT and MRI scans may reveal a spectrum of findings such as enhancing orbital mass, uveoscleral thickening, enlarged extraocular muscles or lacrimal glands, diffuse inflammatory changes, apical lesions, cavernous sinus or paranasal sinuses involvement. Positron emission tomography (PET) scans may also be useful in detecting multi-system inflammatory disease or malignancy. If clinical and imaging results remain ambiguous, orbital biopsy is advised to identify the cause of the orbital inflammation. In the current case, blood tests and orbital imaging indicated an inflammatory process, within the context of multi-system disease and constitutional symptoms such as fatigue, muscle wasting and weight loss. The diagnosis of VEXAS in the current case was ultimately confirmed by bone marrow biopsy and genetic testing.

In summary, VEXAS syndrome should be considered in the differential diagnosis of orbital inflammatory syndromes, particularly in older patients who have multi-organ inflammation and or haematological disorders. With a broad spectrum of clinical manifestations, patients may be under the care of multiple specialists for seemingly unrelated inflammatory conditions, until the unifying diagnosis of VEXAS is confirmed by genetic testing for the UBA1 mutation.

## Data Availability

Data sharing is not applicable to this article as no datasets were generated or analysed during the current study.
